# Dynamic Metabolic Changes in the Human Thalamus at the Transition From Waking to Sleep - Insights From Simultaneous Functional MR Spectroscopy and Polysomnography

**DOI:** 10.3389/fnins.2019.01158

**Published:** 2019-10-30

**Authors:** Mick Lehmann, Andreas Hock, Niklaus Zoelch, Hans-Peter Landolt, Erich Seifritz

**Affiliations:** ^1^Institute of Pharmacology and Toxicology, University of Zurich, Zurich, Switzerland; ^2^Sleep & Health Zurich, University of Zurich, Zurich, Switzerland; ^3^Department of Psychiatry, Psychotherapy and Psychosomatics, Hospital of Psychiatry, University of Zurich, Zurich, Switzerland; ^4^Institute for Biomedical Engineering, ETH Zurich and University of Zurich, Zurich, Switzerland; ^5^Department of Forensic Medicine and Imaging, Institute of Forensic Medicine, University of Zurich, Zurich, Switzerland

**Keywords:** glutamate, electroencephalography (EEG), metabolite cycling, excitability, thalamic reticular nucleus

## Abstract

An important contribution of the thalamus to the transition from wakefulness to sleep is a consistent finding in animal studies. In humans, only little is currently known about the specific role of the thalamus in regulating wake-sleep transitions. Although changes in thalamic blood flow and activity have been reported, the underlying molecular mechanisms have not been investigated. Knowledge about neurotransmitter changes at the wake-to-sleep transition would be indispensable for a better translation of basic animal research findings to humans. Here, we start to fill this important scientific gap. More specifically, we benefit from recent advances in magnetic resonance (MR) spectroscopy, which allow for the non-invasive, local-specific and high-quality detection of naturally occurring metabolite changes in the human brain. We demonstrate in nine young adults able to produce consolidated sleep in the MR spectroscopy scanner, a specific decrease in thalamic glutamate concentration from wakefulness to stage N2 sleep. The magnitude of this decrease was highly correlated with individual N2 sleep duration. When five participants of the original experiment were kept awake in a separate control condition, no decrease in thalamic glutamate levels occurred. The study highlights for the first time in humans that dynamic changes in distinct brain metabolites can be reliably detected at the transition from waking to sleep. The reported methodology to simultaneously acquire functional MR spectroscopy data and neurophysiological signals offers great potential for investigating the molecular mechanisms underlying the transition between and the maintenance of sleep and wake states in humans.

## Introduction

Brain neuronal activity and metabolism are fundamentally different in wakefulness and sleep, particularly in thalamic and cortical networks (for reviews, see e.g., McCormick and Bal, 1997; Vantomme et al., 2019). The electroencephalogram (EEG) in deep non-rapid-eye-movement (NREM) sleep shows a high prevalence of slow waves, which can be quantified by power spectral analysis as slow-wave activity. EEG slow-wave activity (∼ 0.75–4.5 Hz) includes the slow oscillation (<∼1.5 Hz), reflecting widespread cortical alternations between neuronal UP (ON) and DOWN (OFF) periods (Vyazovskiy et al., 2009; Nir et al., 2011), and delta waves (>∼1.5 Hz) that are widely accepted as a homeostatically regulated physiological marker of sleep depth (Achermann and Borbély, 2017). The thalamus and the surrounding thalamic reticular nucleus are thought to make strong contributions to the generation mechanisms of the slow oscillation, delta waves and sleep spindles, which are the EEG hallmarks of NREM sleep in animals and humans (Crunelli et al., 2018; Vantomme et al., 2019).

Consistent with this notion, early positron emission tomography studies in humans revealed that the strongest reduction in regional cerebral blood flow (rCBF) occurred in the thalamus when slow wave sleep was compared to wakefulness (Maquet et al., 1997). Moreover, the changes in rCBF were correlated with EEG delta and spindle (12–15 Hz) frequency activity in NREM sleep (Hofle et al., 1997). More recent functional magnetic resonance imaging (MRI) studies confirmed decreased thalamic activity inferred from arterial spin labeling in NREM sleep when compared to waking (Tüshaus et al., 2017). Intriguingly, simultaneous intracortical and intrathalamic EEG recordings in human epileptic patients demonstrated that the thalamic deactivation occurring at sleep onset typically precedes that of the cortex by several minutes (Magnin et al., 2010). Together with preclinical insights from correlational, lesion/ablation, pharmacological and genetic studies, the available evidence is consistent with an important role of the thalamus for the occurrence of NREM sleep oscillations in the cortex, but also for the transition from wakefulness to sleep (David et al., 2013; Hughes and Crunelli, 2013; Lemieux et al., 2014; Gent et al., 2018a). Nevertheless, the molecular mechanisms controlling the thalamic neuronal firing patterns in wake and sleep states are virtually unknown.

Earlier neurochemical models of brain circuits controlling wakefulness and sleep primarily proposed monoaminergic and cholinergic mechanisms originating in hypothalamus and basal forebrain (Saper et al., 2010; Brown et al., 2012). More recently, it has been argued that these classic neuromodulators merely play a modulatory role, whereas fast acting neurotransmitters such as glutamate (GLU) and γ-aminobutyric acid (GABA) provide the main grid of the wake-sleep regulatory systems (Saper and Fuller, 2017). Indeed, it has long been proposed that a decrease in GLU and an increase in GABA function are fundamental for sleep-related behaviors (for an overview, see Jones, 2011). Interestingly, modafinil, a wake-promoting agent, increases GLU in thalamic areas (Ferraro et al., 1997). While subregions of the thalamus have emerged as essential hubs for controlling cortical activity and behavioral state (Giber et al., 2015; Liu et al., 2015; Gent et al., 2018b), the exact roles of GLU and GABA, the main excitatory and inhibitory neurotransmitters, in sleep-wake regulation remain unclear.

Knowledge about dynamic changes in these neurotransmitter systems in humans at the wake-to-sleep transition is currently lacking. To start filling this scientific gap, we used recent methodological advances that allow for the non-invasive detection of naturally occurring metabolite concentrations with high data quality in circumscribed areas of the human brain. We acquired functional MR spectroscopy data with high temporal resolution simultaneously with polysomnography, to objectively define the wake-to-sleep transition in healthy adults. Based on previous data in rats (Dash et al., 2009), we hypothesized that the GLU level in the thalamus would decrease from wakefulness to sleep and that the magnitude of this decrease may be associated with the depth of sleep.

## Materials and Methods

### Participants

Fifteen adults between 19 and 24 years of age were recruited via advertisements at the University of Zurich. All participants had previous experience with MR studies, were familiar with staying in the scanner for more than 45 min and sleeping on their back in the constrained scanner environment. None fulfilled any of the following exclusion criteria: history of psychiatric/neurological diseases, sleep-wake abnormalities, drug abuse, concurrent medication use, cardiovascular disease, MR exclusion criteria, and pregnancy. No traveling across time zones was allowed for at least 6 weeks before the study. Subjects had to refrain from alcohol, caffeine and sports activities during 24 h prior to the experiment. The Ethics Committee of the Canton of Zurich approved the study. All subjects gave written informed consent before screening.

### Experimental Procedure

One week prior to the experimental night, participants were instructed to keep a regular sleep-wake schedule according to their individual habitual sleep-wake times. Compliance was verified with self-reported sleep logs and wrist motor actigraphy and light sensors (MotionWatch 8, CamNtech Ltd., Cambridge, United Kingdom). In the night prior to the assessment, the subjects’ average sleep duration across the pre-study week was reduced by 2 h to slightly elevate sleep pressure. Actigraphic data were controlled at the beginning of each experimental session, to ensure compliance with the study instructions.

On the night of the MR spectroscopy and sleep recordings, subjects arrived ∼ 3 h before their usual bedtime at the MR center of the Psychiatric Hospital Zürich for the application of the electrodes for polysomnography. The experimental set-up included 32 EEG channels according to an extended 10–20 system (Brain Products GmbH, Gilching, Germany), electrooculogram (EOG), submental electromyogram (EMG), and electrocardiogram (ECG). The MR protocol started with a structural MRI scan around 2 h before the subjects’ habitual bedtime, to avoid sleepiness in the scanner. Participants were asked to stay awake for at least 20 min during initial MR spectroscopy scanning, to collect sufficient data during wakefulness. Afterward, they were permitted to sleep. The MR spectroscopy and polysomnographic measurements were continued for as long as the volunteers were able to remain in the MR scanner without gross movements. Six study participants were unable to sleep (*n* = 2) or slept for less than 15 min (*n* = 4). They were excluded from data analyses, resulting in a final study sample of five females and four males (mean age: 22.3 ± 2.2 [SEM] years).

To control for diurnal or non-sleep-related temporal changes in metabolite ratios during the prolonged measurement period, all participants who were able to sleep in the scanner for longer than 15 min in sleep stages N2 and N3 were invited for a second recording. This additional experimental condition followed the same procedures as described above, yet the volunteers were required to stay awake as controlled by polysomnography for the entire duration of MR spectroscopy scanning.

### MR Spectroscopy Data Acquisition

**FIGURE 1:**
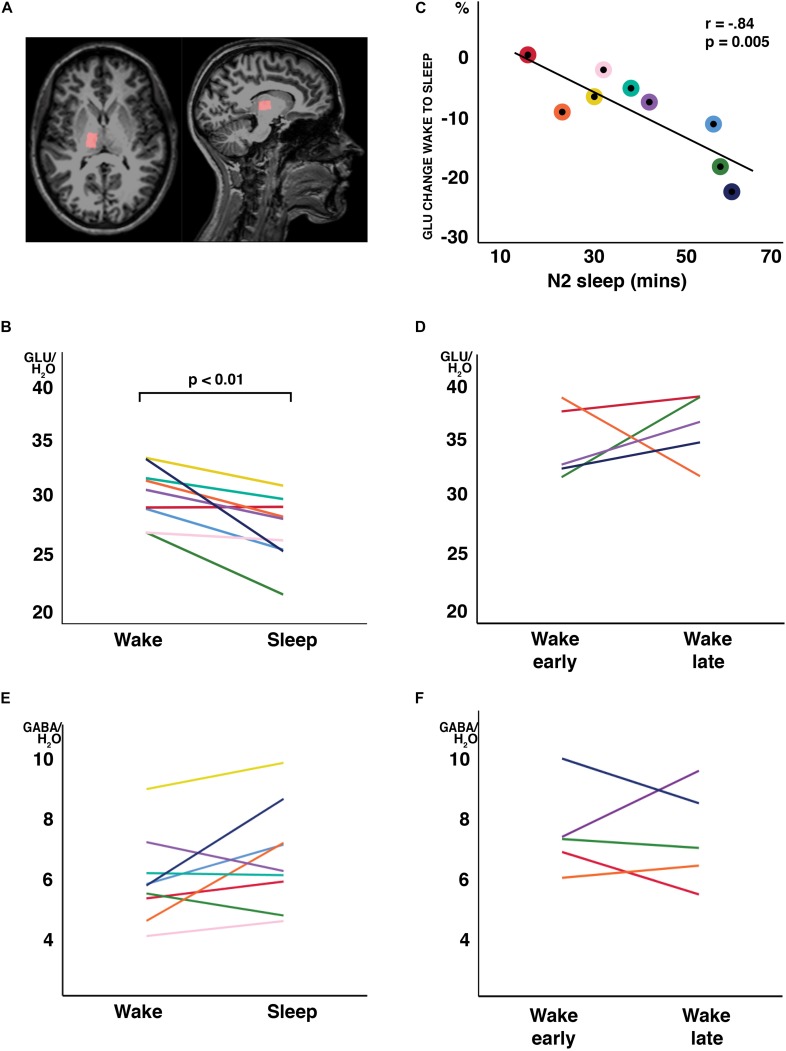
Changes in water-scaled glutamate (GLU) ratios from wakefulness to N2 sleep. **(A)** MR spectroscopy voxel placement in the thalamus. **(B)** GLU levels decreased from wakefulness to sleep in 8 of 9 study participants (arbitrary units; *p* < 0.01, two-tailed, paired *t*-tests) **(C)** The change from wakefulness to sleep was negatively correlated with N2 sleep duration. Individual participant’s data have the same color code in panels **(B,C)**. **(D)** When a subset of participants stayed awake during an equivalent recording period at the same time of day, no changes in GLU were observed. No changes were observed in GABA ratios, neither from wakefulness to sleep **(E)** nor during the wake control condition **(F)**. Each individual participant’s data have the same color code in panels **(B–F)**.

^1^H-MR spectroscopy measurements were performed on a Philips Achieva 3T whole-body scanner equipped with a 32-channel receive-only phased-array head coil (Philips Healthcare, Best, The Netherlands). Non-water suppressed, PRESS-localized ^1^H-MR spectroscopy spectra in the thalamus (Figure 1A) were obtained using the metabolite cycling (MC) technique (Hock et al., 2013). This technique allows for the correction of frequency and phase changes caused by system instabilities (e.g., B0-field drifts, blood or cerebrospinal fluid flow, patient movements) between each single acquisition. In addition, this method enables a flexible selection of acquisitions from different time points for a retrospective averaging within the scan session based on the sleep states defined by the polysomnographic analyses. The PRESS localization sequence parameters were chosen as follows: 2500 ms repetition time, 32 ms echo time and 2000 Hz spectral bandwidth and was combined with inner-volume saturation (Edden et al., 2006).

### Polysomnographic Recording During MR Spectroscopy Scanning and Artifact Correction

Wakefulness and sleep during scanning were confirmed by polysomnography as described above using an MR-compatible polysomnographic recording system (BrainAmp, BrainCap and BrainAmp ExG MR devices and electrodes; Brain Products GmbH, Gilching, Germany). Impedances of all electrodes were kept below 10 kΩ. The bioelectric data were synchronized with the scanner clock, sampled at 5 kHz (filtered between 0.01 and 200 Hz) and referenced to the vertex (Cz).

### Sleep EEG Correction and Sleep-State Scoring

Offline EEG data correction was performed using BrainVision Analyzer 2.0 (Brain Products, Gilching, Germany). Removal of MR spectroscopy artifacts was based on the average artifact subtraction method, whereas 21 MR spectroscopy artifact intervals were used for sliding average (Allen et al., 2000). Subsequently, data were corrected with a standard cardioballistogram (CB) artifact template correction (Allen et al., 1998). The cardioballistic artifact template was determined from the cardiac waveform recorded by the ECG channel. The CB artifact to be subtracted was defined by a moving average over 21 cardiac periods. Both, CB and MR artifact correction routines are pre-implemented in the software. The EEG data were high-pass filtered at 0.3 Hz and low-pass filtered at 35 Hz (48 dB/octave) and subsequently down-sampled to 512 Hz for the scoring of waking and sleep states. After MR spectroscopy and CB artifact correction, the polysomnographic recordings were scored offline in 30-s epochs by two independent raters according to standard criteria (Iber et al., 2007).

### MR Spectroscopy Analyses

Each single MR spectroscopy acquisitions was phase corrected and frequency aligned exploiting the advantages of the non-water suppressed acquisition scheme before acquisitions scored as the same sleep state were averaged. After averaging, eddy-current correction (Klose, 1990) was applied using the unsuppressed water signal to reduce induced line-shape distortions. Additionally, a Hankel singular value decomposition water filter was applied to remove the remaining water peak in the resulting metabolite spectrum (Cabanes et al., 2001).

For quantification of spectral data, LCModel (Version 6.3) with a set of simulated basis spectra, consisting of 18 metabolites, was used (Provencher, 1993). For each metabolite, the ratio to the unsuppressed water signal was calculated.

Based on the polysomnographic recordings, single MR acquisitions were classified into wakefulness and NREM stages, N1, N2, and N3. Signal-to-noise-ratio (SNR) and full-width-at-half-maximum of the spectra according to the LCModel output were compared between categories (i.e., wakefulness and sleep states).

### Statistical Analyses

Metabolic ratios between wakefulness and sleep were compared using paired *t*-tests. The relationship between sleep variables and changes in metabolic ratios were analyzed using Pearson’s product-moment correlation coefficients. A Bonferroni-corrected probability of *p* = 0.05 was set as the significance threshold, yet the uncorrected *p*-values are reported.

## Results

Actigraphic data to control the pre-experimental study instructions were verified at the beginning of each experimental night.

The “sleep” experiment started at 9.56 pm ± 56 min. Following the pre-sleep wake scan and once permitted to fall asleep, the participants took on average 10.3 min (SD = 7.4 min) to produce N2 sleep and maintained 79.7 min (SD = 20.8 min) of sleep in the scanner (range: 37 - 115 min). Sleep was composed of roughly 20% N3, 57% N2 and 13% N1 sleep, while wakefulness after sleep onset equaled approximately 10% (Table 1). No REM sleep occurred.

**TABLE 1:** Visually scored sleep variables.

**Sleep variable**	**Duration (min)**	**Percentage of TST (%)**
Wakefulness before sleep	29.1 ± 8.4	n/a
Sleep latency	10.3 ± 7.4	n/a
Total sleep time (TST)	79.7 ± 20.8	n/a
N1 sleep	9.1 ± 5.6	12.8 ± 9.0
N2 sleep	45.4 ± 15.5	57.3 ± 14.4
N3 sleep	18.2 ± 17.5	20.6 ± 14.9
REM sleep	0.0 ± 0.0	0.0 ± 0.0
WASO	7.0 ± 5.2	9.4 ± 6.6

*Mean values ± SD are reported (*n* = 9). Sleep latency: time after permission to fall asleep until first occurrence of N2 sleep, defining sleep onset. REM sleep, rapid-eye-movement sleep; WASO, wakefulness after sleep onset.*

Analyses of the metabolite changes indicated that the SNR differed significantly between waking and sleep, when “N3” sleep was included in the category “sleep” (*t*_8_ = 7.20, *p* < 0.001, d = 1.01). This rendered the metabolite ratios non-comparable between wakefulness and sleep. The SNR did not differ when ratios were compared between wakefulness and N2 sleep (*t*_8_ = 0.68, *p* = 0.541).

In all participants but one, the thalamic GLU concentration decreased from wakefulness to N2 sleep (*t*_8_ = −3.74, *p* = 0.006, d = 1.25; Figure 1B). During the 2-h recording period, the decrease in GLU was tightly inversely correlated with the duration of N2 sleep (*r* = −0.84; *p* = 0.005; Figure 1C). Interestingly, the single subject not showing a reduction in thalamic GLU levels, only produced 17 min of N2 sleep in the MR scanner (Figure 1C; “red” participant). Apart from GLU, no other metabolite, including GABA (Figure 1E), showed a difference between wakefulness and sleep (*p* > 0.117; Table 2).

**TABLE 2:** Water-scaled metabolite concentrations in wakefulness before sleep and during sleep.

**Metabolite**	**Wakefulness**	**Sleep**	***t*-value**	***p*-value**
Glutamate	30.1 ± 2.2	27.4 ± 2.6	–3.74	0.006
Glutamine	6.6 ± 3.0	7.4 ± 3.2	0.82	0.44
Glutamate + glutamine	36.7 ± 3.9	34.8 ± 4.5	–1.76	0.12
γ-Aminobutyric acid	5.8 ± 1.4	6.5 ± 1.7	1.74	0.12
Aspartate	6.1 ± 1.5	5.2 ± 1.9	–1.71	0.31
N-acetyl-aspartate	39.1 ± 2.5	38.8 ± 3.1	–0.51	0.62
Myoinositol	16.1 ± 1.3	16.2 ± 2.0	0.25	0.81
Glutathione	2.2 ± 0.6	2.2 ± 0.7	–0.10	0.92

*Mean values ± SD in arbitrary units are reported (*n* = 9). *t*- and *p*-values refer to two-sided, paired *t*-tests.*

To determine whether the observed changes in thalamic GLU concentration were sleep related or merely reflected diurnal or temporal changes in metabolite ratios, seven participants of the “sleep” experiment completed a control recording at the same time of day, but in the absence of sleep. The presence of wakefulness during the entire duration of functional MR spectroscopy scanning was controlled by polysomnography. Five volunteers were able to stay awake during the entire control measurement (89.3 ± 21.5 min). Confirming that sleep is required for the observed decrease in GLU at the sleep-to-wake transition, no reliable changes in GLU (Figure 1D) nor in any other metabolite ratio [including GABA (Figure 1F); other data not shown] were observed when subjects stayed awake.

## Discussion

The present study provides first experimental evidence for dynamic changes in thalamic GLU concentration at the transition from wakefulness to sleep in humans. The unique data set required the ability to average individual functional MR spectroscopy acquisitions based on the polysomnography-defined occurrence of wakefulness and sleep states. These methodological innovation enabled the reliable quantification of minute metabolic changes in the glutamatergic system between wakefulness and sleep.

At the transition from waking to sleep, a specific decrease in GLU was observed whereas other metabolites did not change. This suggests that our finding is not biased by a general reduction in the water signal, which was used for the scaling of the metabolite values. Moreover, the decrease in GLU was stronger in participants reaching more stable NREM sleep. This observation is in line with previous observations in rat cerebral cortex showing that the rate of decrease in GLU varies with sleep depth and increases when animals are awake (Dash et al., 2009). The latter indicates that changes in glutamatergic metabolism are not mainly driven by circadian mechanisms. Of note, Dash and colleagues studied sleep-wake related and diurnal changes in GLU in rats and their findings may not be transferred directly to humans. In the present study, a circadian contribution to the level of GLU in the human thalamus was not assessed. Moreover, while the removal of scanner artifacts from the EEG allowed the reliable scoring of wakefulness and sleep states, a reliable quantitative analysis of the EEG signals was not possible. Nevertheless, the wake control experiment corroborated the observation in rats that the cerebral GLU levels do not decrease in reliable manner in the absence of sleep. Substantial preclinical evidence supports important roles of thalamic neurons and thalamo-cortical networks in EEG NREM sleep oscillations and the control of behavioral states (wake *vs.* sleep) (Crunelli et al., 2018; Gent et al., 2018b; Vantomme et al., 2019). The present human data are consistent with the hypothesis that an elevated thalamic GLU concentration promotes the tonic, single-spike activity of thalamo-cortical neurons and the thalamic reticular nucleus typical for wakefulness, whereas reduced thalamic GLU levels facilitate the occurrence of EEG NREM sleep oscillations and consolidated sleep. This conclusion may also be consistent with computational modeling (Destexhe et al., 1994) and *in vitro* slice electrophysiology data (Cox and Sherman, 1999).

Even though 6 out of 9 participants showed an increase in GABA concentration from wakefulness to sleep, the change was not significant. The reliable detection of GABA with MR spectroscopy in small voxels is generally difficult because of the relatively low abundance of GABA and the spectral overlap with signals from more abundant metabolites. We found no correlation between the wake-sleep related changes in GLU and GABA ratios (*r* = −0.405, *p* = 0.280; data not shown). In addition, similar to our study, administration of modafinil to promote wakefulness in rats specifically increased GLU release in ventromedial and ventrolateral thalamic nuclei while the release of GABA remained unaffected (Ferraro et al., 1997). Although the physiological significance of pharmacologically induced alterations in wake and sleep states has to be interpreted with caution, the present and previous data indicate that merely altering the balance between GLU and GABA may be sufficient to switch the system between wakefulness and sleep. It also has to be kept in mind that the participants in the present study were slightly sleep deprived. Future work may address the question whether differences in homeostatic sleep pressure affect the rate at which brain GLU levels change at the transition from waking to sleep.

Similar to GABA, no changes in thalamic GLX were found at the wake-to-sleep transition. The GLX signal captures the combination of GLU and glutamine which is the biosynthetic precursor of both, GLU and GABA. Virtually opposite changes in GABA and GLU concentrations from waking to sleep may, thus, obscure potential changes in GLX resulting from a decreased GLU level. Interestingly, a strong relationship between right thalamic GLX levels and disturbed sleep was reported in patients with restless legs syndrome (Allen et al., 2013). In that study, however, MR spectroscopy and polysomnographic recordings were not conducted simultaneously such as in the current work.

## Conclusion

In conclusion, this study provides first experimental evidence in humans that dynamic metabolic changes can be measured at the transition from wakefulness to sleep. Because of the relatively low sample size, the findings should be qualified as preliminary. Nevertheless, apart from their importance for informing the search for molecular mechanisms underlying human sleep-wake regulation, these insights also have implications for the interpretation of studies using resting state MR spectroscopy as a diagnostic tool for sleep-wake and neuropsychiatric disorders (Allen et al., 2013; Moriguchi et al., 2019). Drowsiness and (N2) sleep likely occur in many individuals undergoing MR scanning (Tagliazucchi and Laufs, 2014). Given the evidence provided here, that GLU concentrations differ between wakefulness and N2 sleep, control for changes in vigilance state will be required for the careful interpretation of future MR spectroscopy findings. Moreover, it will be of great interest to investigate whether levels of GLU are globally decreased when the awake brain enters sleep or whether this decrease is restricted to central hubs subserving sleep-wake control such as the thalamus.

## Data Availability Statement

All datasets generated for this study are included in the article/supplementary material.

## Ethics Statement

The studies involving human participants were reviewed and approved by the Ethics Committee of the Canton of Zurich. The patients/participants provided their written informed consent to participate in this study.

## Author Contributions

NZ and AH developed the functional MR spectroscopy sequence and the MR spectroscopy data post-processing. ML and AH designed the experiment. ML, AH, and NZ carried out the data collection. ML analyzed the EEG data for sleep scoring. ML and NZ analyzed the MR spectroscopy data. All authors wrote, discussed, and approved the manuscript.

## Conflict of Interest

The authors declare that the research was conducted in the absence of any commercial or financial relationships that could be construed as a potential conflict of interest.
